# Finding clues in the p53 maze: an interview with Karen Vousden

**DOI:** 10.1242/dmm.035675

**Published:** 2018-07-27

**Authors:** Karen Vousden

**Affiliations:** Karen was appointed Commander of the Order of the British Empire (CBE) in 2010 and Foreign Associate of the US National Academy of Sciences in May 2018. She is group leader at the Francis Crick Institute in London and Chief Scientist at Cancer Research UK. She is also a Fellow of the Royal Society (2004)

## Abstract

Karen Vousden is an internationally renowned cancer scientist whose contributions to solving the p53 puzzle are changing the way we think about this important tumour suppressor. She has been honoured with many prizes and elected memberships throughout her career, and was appointed Commander of the Order of the British Empire (CBE) in 2010 and Foreign Associate of the US National Academy of Sciences in May 2018. Karen is an approachable and diligent scientist, a respected mentor and an inspirational role model. She is currently a group leader at the Francis Crick Institute in London and Chief Scientist at Cancer Research UK (CRUK). In this interview, Karen talks about the mentors that inspired her, the discoveries that brought about her interest in cancer metabolism, and her interests outside the lab.

Karen Vousden's research focuses on the many functions of p53 in cancer and beyond. Her scientific journey in the field of genetics started with undergraduate and graduate work on transfer RNA (tRNA) suppression with Lorna Casselton at the University of London, UK. She studied Ras and viral oncogenes during her postdoctoral training with Christopher Marshall and Douglas Lowy. The interactions between viral oncogenes and mammalian tumour suppressors drew her into the field of p53. Her group (in parallel with others) discovered the p53 targets *PUMA* and *TIGAR*, the latter of which spurred her interest in cancer metabolism.


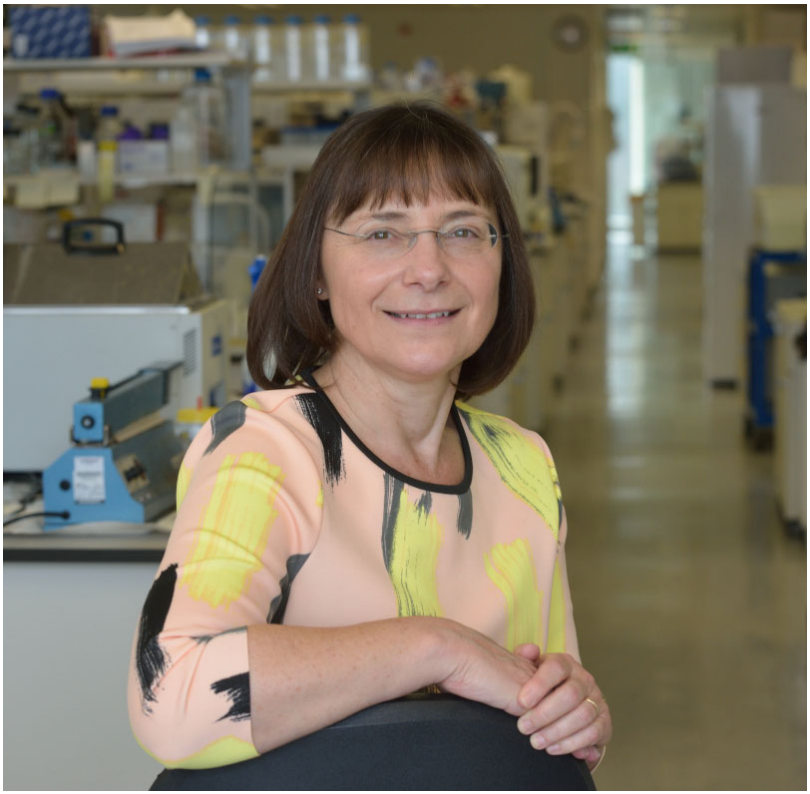


**Looking back at your career, do you think that your younger self would be surprised by your career path?**

Yes, I think that would be fair to say. I do not know if I would ever have predicted that I would have the job that I have now. I am very privileged and slightly bemused by how I got to be so lucky.

I did start liking science very early on and I would be satisfied with just doing bench work. I always enjoyed being in the lab, thrilled by the discovery and the exploration. In my younger days, I never thought that I would have the privilege of running a lab, or to have my new job at CRUK, where I am able to help so many other scientists do what they do so well. So, yes, it has been both wonderful and astonishing.

**Your career has indeed been inspiring. Which key people inspired you the most?**

I think my key inspiration were people who had faith in me when I did not have faith in myself. That's what we all need, isn't it? My first postdoc supervisor, Chris Marshall [Christopher Marshall, who sadly died in 2015; Institute for Cancer Research, London, UK], was a fabulous inspiration. He showed me what excitement and what fun science can really be. We were doing important work, right at the beginning of discovering oncogenes and identifying *Ras* mutations in human cancers. Chris not only taught me how important it is to work on a big question, but also to have fun doing it. He encouraged me to believe that science was something that I can do, and that I should not compromise on how far I wanted to go. Then my second postdoc advisor, Doug Lowy [Douglas R. Lowy, National Cancer Institute (NCI), MD, USA], was endlessly supportive and encouraging, despite my many failures in his lab. Doug also gave me his car to drive when I first showed up as a postdoc in his lab – there's trust for you!

Another huge influence was George Vande Woude [currently holding an emeritus position at Van Andel Institute, MI, USA], who recruited me to be a group leader at the ABL [Advanced BioScience Laboratories] Basic Research Program, part of the NCI in Frederick, Maryland. At one point George stepped down from the ABL to take up a bigger role at the NCI [as special advisor to the Director, and later as Director, of the Division of Basic Sciences at NCI], and he encouraged me to take over as interim Director. ABL was a highly regarded and prestigious institute; I was very young and would never ever have dreamt of applying for such a role. But George had faith in me and so I was given this fantastic opportunity to be a scientific leader as well as conducting my own research. This experience at ABL gave me the confidence that I could be a leader and led to my being offered the job as Director of the CRUK-Beatson Institute in Glasgow.

It is a recurring theme, but it was the people that were willing to both push me while holding my hand at the same time that have been most inspirational to me personally. And, of course, every other fantastic scientist around the world.

“It was the people that were willing to both push me while holding my hand that have been most inspirational to me personally. And, of course, every other fantastic scientist around the world.”

**Going back to science itself: we have known about p53 for about 40 years now, and are still learning about its many functions. Can you tell us what brought about your interest in this important tumour suppressor?**

Yes, p53 was first described in 1979, so it will be 40 years next year. Following my nose brought me to the field. While with Chris [Marshall], I worked on Ras-mediated transformation. We hardly knew anything about it as, at the time, people were still debating whether or not cancer had a genetic basis. Discovering oncogenes was a huge leap. Because I was working on Ras, I looked for a second postdoc in a Ras-focused group. I went to work with Doug Lowy at the NCI, who worked on both Ras and papillomaviruses [Doug Lowy was instrumental in the development of the HPV vaccine]. In the first few days in his lab in 1985, Doug asked me which area I wanted to work in. I remember calling Chris, because he was my buddy, asking him what I should do. Chris said, “We sorted Ras out, we know everything there is to know about it. Why don't you go work on papillomavirus?” I find this quite funny because we still don't know everything there is to know about Ras, let alone p53.

Taking Chris's advice, I chose to work on papillomavirus. But I wasn't a virologist, so I did what I had previously done with Ras – I looked for transforming activity in papillomavirus. This allowed me (along with several other independent scientists) to identify *E6* and *E7* as oncogenes in the papillomavirus genome.

Ed Harlow and Peter Howley [both at Harvard Medical School, MA, USA] showed that these two viral proteins interact with cellular proteins, and Peter showed that one of the major cellular binding partners of E6 is p53. I was eager to get back to studying mammalian genes, and p53 was just perfect. Moshe Oren and Varda Rotter [both at the Weizmann Institute of Science, Israel], David Lane [currently at A*STAR, Singapore] and Arnie Levine [Arnold Levine, currently at the Institute for Advanced Study, NJ, USA] were doing brilliant work on p53 at the time. I went to one of the very first p53 meetings at the Marie Curie Institute in Oxted in Surrey. At that time the field was very small and it was such a friendly bunch. So I realised this was the area of research for me. With my small lab we started to expand on how the E6-p53 interaction worked and then we very quickly switched to work exclusively on p53's functions.

This was in the mid-80s, the first decade of p53 research, and we were all a bit confused about what it does. Soon afterwards came the realisation that p53 was not an oncogene, but a tumour suppressor that was mutated in many cancers.

Because mutant p53 has such a strong cancer-related phenotype and p53-null mice initially looked fairly normal, people got into the mind-set that all p53 did was inhibit cancer. It was an exciting area to work in, especially with the understanding of mutant p53 functions over and above wild-type p53, so the field spent a lot of energy researching p53 in the context of cancer. I think at the time we missed some of the other, subtler, activities of p53, which are also extremely important. p53 is involved in many stress responses, which obviously have a big impact on cancer, but it also has a big impact in many other areas of health and disease.

“It was an exciting area to work in, especially with the understanding of mutant p53 functions over and above wild-type p53, so the field spent a lot of energy researching p53 in the context of cancer. I think at the time we missed some of the other, more subtle, activities of p53…”

Over the past 10 years, many of us have started to look beyond oncogenic stress to things like metabolic or physiological stress – overeating, starvation, etc. We are now beginning to understand that p53 plays a role in quite a broad range of pathologies and in maintaining homeostasis. There have been indicators of this through the years – I remember Arnie Levine's fabulous experiment where he put mice into a little tube to restrict their movement, which stressed them. He then showed p53 induction. I thought that was a very interesting experiment. There is a lot of discussion on how bad stress is for people, and it looks like stress induces p53, which has all kinds of consequences.

**As we just discussed, we are constantly learning about new functions of p53. Can you tell us more about how new research in gain-of-function *TP53* mutations is opening new therapeutic windows?**

It is an unbelievably attractive observation that mutations in p53 seem to be selected for in cancers. Many cancers retain high levels of expression of these mutant proteins and several depend on the continuous expression of mutant p53. So mutant p53 must be the perfect therapeutic target because, by and large, normal cells don't express it. I think it is a bit frustrating that we still haven't quite managed to use this to help cancer patients.

The gain-of-function mutations are interesting because they are clearly real, but they are quite difficult to chase, as different p53 mutants have different functions in different tumours. A certain p53 mutation may promote metastasis in pancreatic cancer, but does not have the same effect in a lung tumour. We still do not understand exactly what the important functions of these gain-of-function mutants are and why their activities seem to be tissue specific. Until we understand exactly what they are doing, it will be hard to target them.

For a while, we were all working on the hypothesis that mutations in p53 alter the protein to enable the gain of neomorphic functions. But there is an alternative suggestion, which is that p53 mutants lose only some of their wild-type activities, while retaining others. In general, the tumour-derived p53 mutants lose the ability to kill or eliminate cells – which wild-type p53 can do – but some of them retain some p53 activities that are important for adaptation to metabolic stress. So those tumour-derived mutant p53 are able to support survival, but have lost the ability to kill or inhibit the proliferation of cells. That is not really a gain of a new function, but rather a selective retention of only some of the wild-type p53 activity.

**The more we learn about p53, the more questions open up. Did the discovery of these adaptive mutant forms of p53 spur your interest in cancer metabolism?**

It was the other way around, actually. We became interested in metabolism because we described the p53 target gene *TIGAR*, which functions to regulate glycolysis and the pentose phosphate pathway in a rather complicated way. So a metabolic gene is clearly a target of p53. We identified *TIGAR* in an expression array screen years and years ago. I had a fabulous postdoc who was incredibly rigorous in the identification of p53 target genes. In the end, he narrowed the screen's hits down to three genes that he seriously believed were bona fide p53 targets. One of them was *PUMA*, which we published, one was ribonucleotide reductase, which was described by other people, and the third was *TIGAR*.

We were confident that *TIGAR* is a p53 target gene, so we tried to understand its function. We discovered it had bisphosphatase activity and could help to regulate glycolysis, in a way that supported antioxidant defence. So *TIGAR* seems to protect cells from oxidative stress and ROS-induced damage and cell death, and made us seriously consider the role of p53 in regulating metabolism. That was also our first clue that p53 could protect cells in conditions of stress rather than killing them, and we now know of several different mechanisms by which p53 can do this. The idea that some cancer-associated p53 mutants can retain these functions came much more recently.

**Recent work on cancer metabolism from your group showed that a controlled diet can alter the progression of cancer in mouse models. Are we close to prescribing a defined diet as treatment for cancer patients?**

I hope so! We actually took a very simplistic approach in our mouse studies, as we limited the availability of a specific amino acid in food. Our work focused on serine and glycine deprivation, but other amino acids have been shown to affect tumour development and progression. We are now busy designing clinical trials to see whether we can use amino-acid-defined diets in association with either conventional chemotherapy or maybe even with new treatment options. I do not think that we would expect patients to go on amino-acid-depleted diets for life; I think that might be asking too much. But as an adjunct to a conventional cancer treatment regime, where we can show what type of genetic makeup of the tumour would be particularly susceptible to these kinds of interventions, it is realistic. We could design a precision nutrition regimen based on the characteristics of the tumour and the type of therapy.

**Nutrition is something the general public can understand**

Yes. But we are trying to avoid misinterpretation. We are trying to be very clear about the mechanisms behind these effects and that these findings are based on hard science. It is sometimes easy for the press to misinterpret or oversimplify results. We do not want people to self-medicate with a diet based on selected conclusions and extrapolations from our publication. We are working hard to keep such interventions controlled and we want to be absolutely sure that we have an evidence-based protocol before moving into clinical use.

**Do you think such a defined diet would be feasible for cancer prevention, or would this preferably work as a therapeutic support for people who already have cancer?**

The type of diet we are envisioning, which is serine- and glycine-depleted, would involve eating no protein at all and then supplementing with a defined amino acid mix. From a wellbeing and quality of life point of view, such a diet does not sound attractive to me. While technically some controlled diets may work in reducing the risk of cancer, I think we will need to find a cleverer way of tweaking the availability of certain amino acids instead of eliminating them from a person's diet. I just do not think that would be any fun.

So, realistically, I think such a dietary intervention would be best suited for boosting the effectiveness of therapy in patients who are already being treated for cancer.

**The revived interest in metabolic aberrations in cancer has led scientists back to textbooks and seminal papers from decades past. How important is it to rely on the legacy of such early work in your field?**

I think we have all realised how astonishingly wonderful all that classic literature is. It is incredible how scientists in the 1930s, 40s and 50s managed to get to the right answers, using what we would now consider completely medieval approaches. It is a tribute to how clever they were. I would say that the majority of the classic textbook pathways that were established 50-60 years ago are absolutely correct. Obviously, we are now layering on further complexities, as we understand more details and more nuances, but the basic bones of what was set up all those years ago is completely right. I think this makes the field very interesting, because we did not have to start from the beginning – we were able to build on the foundations of the existing data.

**It is remarkable, I agree. Although, for many students, learning all these pathways can be a bit of a nightmare, maybe not quite the definition of fascinating?**

I, like many undergraduates, chose not to bother to learn them. And now that's come back to haunt me! [Laughs]

**Aside from your hands-on research, you are also Chief Scientist for Cancer Research UK. Can you tell us more about this role?**

The role is mainly advisory, because CRUK has an outstandingly fantastic team overseeing research and innovation – I am just trying to support them. I try to take a broader view on where CRUK invest their funds in the basic and translational research arena and make sure that the best scientists become part of the CRUK team and that they get everything they need to accelerate progress. I also try to identify new and exciting areas in which we may encourage people to venture. CRUK also has a Chief Clinician, Charlie Swanton, and we work together to help steer all aspects of CRUK research from bench to bedside. So, it is a great job – to help to decide how to spend the money that our talented fundraisers raise for cancer research. It is very rewarding and a great deal of fun to enable others to do the amazing research that they do.

**So this job gives you the opportunity to identify new areas of cancer research, both basic and translational?**

Obviously, there is a limited pot of money, and we can always find more things to spend the money on than there are available funds. But we do think hard about how we can provide new funding mechanisms for researchers and to try to bring new people to the field. For example, immunologists who have never thought about steering their research towards cancer-related problems, or experts in the physical sciences, such as engineers and mathematicians. Can they turn their experience and expertise towards problems in cancer? I do not know much about computational approaches and artificial intelligence, but I know enough to understand that they are important and hold much promise. We are trying to invite experts in these areas to become engaged in solving problems in cancer research. When we describe to data scientists the enormous wealth of information available to cancer researchers, they get very excited – which is great fun for me.

**As you have been in leadership roles for some time now, you have had the chance to mentor a number of people. Is there any advice you would like to share with your younger colleagues?**

Science requires hard work and, to some extent, luck. I try to tell my PhD students and postdocs that they really need to be absolutely inspired and in love with science. Because, although it can be a difficult path, for those who really love it, there is nothing better. I think research is as much a calling as it is a profession. Hard work, but also incredibly rewarding. I try to encourage people to understand that, if science is what they absolutely want to do, they should not give up when things get tough. Just stick with it. I have never seen anyone who really tried not to succeed in moving his or her career towards an independent position. I guess many people lack confidence, which takes us back to what I said at the beginning. Most people have much more talent and ability than they think they do, and the best thing I can do as a mentor is try to make them feel confident and unafraid.

**If you were not a scientist, what career would you choose?**

That is an interesting question. I have not thought much about it, because I have never not wanted to do science. If I could not be a scientist, I think I might like to be an architect. I had a few experiences designing buildings, both at work and at home. I loved doing it and became very involved in the process, I guess; more than any ‘normal’ human being may want to. I feel that I have some ability to translate a plan, an idea, into a building. It is interesting, because I am not particularly artistic, I can't draw or paint, but I can visualise what something is going to look like from a plan.

**And our final question: what do you enjoy doing outside of the lab?**

The main things I enjoy are travel, for which being a scientist is great, and I like hiking and walking. We spent 2 weeks in the Himalayas last November, trekking around Annapurna and Everest. We've also hiked in Peru and Chile – each of these trips was astonishing. Being in Scotland [during her tenure as Director of the Beatson Institute] was great for spending some time in the hills. London is a less ideal base, but we still have our house in Scotland, and spend time there quite often. My plan is to spend several months at a time exploring new countries after I retire – which I'm not planning on doing any time soon.

This article is part of a special subject collection ‘Cancer metabolism: models, mechanisms and targets’, which was launched in a dedicated issue guest edited by Almut Schulze and Mariia Yuneva. See related articles in this collection at http://dmm.biologists.org/collection/cancermetabolism.

